# Genomic insights of *Klebsiella pneumoniae* isolated from a native Amazonian fish reveal wide resistome against heavy metals, disinfectants, and clinically relevant antibiotics

**DOI:** 10.1016/j.ygeno.2020.09.015

**Published:** 2020-11

**Authors:** Louise Cerdeira, Daniel F.M. Monte, Bruna Fuga, Fábio P. Sellera, Ingrith Neves, Larissa Rodrigues, Mariza Landgraf, Nilton Lincopan

**Affiliations:** a Department of Clinical Analysis, Faculty of Pharmaceutical Sciences, University of São Paulo, São Paulo, Brazil; b Department of Infectious Diseases, Central Clinical School, Monash University, Australia; c One Health Brazilian Resistance Project (OneBR), Brazil; d Department of Food and Experimental Nutrition, Food Research Center, Faculty of Pharmaceutical Sciences, University of São Paulo, Brazil; e Department of Microbiology, Instituto de Ciências Biomédicas, Universidade de São Paulo, São Paulo, Brazil; f Department of Internal Medicine, School of Veterinary Medicine and Animal Science, University of São Paulo, São Paulo, Brazil

**Keywords:** Enterobacterales, ESBL, Food, *qnrE1*, Resistome

## Abstract

A multidrug-resistant CTX-M-15-producing *Klebsiella pneumoniae* (KpP1 strain) was isolated from a native Amazonian fish (*Brachyplatystoma filamentosum*) at the Brazilian Amazon. The strain was identified by MALDI-TOF. The genome was extracted, purified and a Nextera DNA Flex library was prepared and sequenced by Illumina platform. The sequenced genome was *de novo* assembled using Unicycler and *in silico* prediction accomplished by curated bioinformatics tools. The size of the genome is 5.6 Mb with 5715 genes. Whole-genome sequencing analysis revealed the presence of wide resistome, with genes conferring resistance to clinically relevant antibiotics, heavy metals and disinfectants. The KpP1 strain was assigned to the sequence type ST3827, KL111 (*wzi*113) and O3b locus. Native freshwater fish sold in wet markets of the Amazonian region could be an important vehicle for transmission of multidrug-resistant bacteria to humans. This study may give genomic insights on the spread of critical-priority WHO pathogens in a One Health context.

## Highlights

• A multidrug-resistant *Klebsiella pneumoniae* was isolated from a native Amazonian fish.
• Genomic analysis revealed that *K. pneumoniae* belonged to ST3827, KL111 and O3b locus.
• A wide resistome against heavy metals, disinfectants, and relevant antibiotics was predicted.
• ESBL production was associated with the *bla*_CTX-M-15_ gene carried on an IncFII(K) plasmid.
• Native freshwater fishes could be a vehicle for transmission of critical-priority pathogens.

## 1 Introduction

The Brazilian Amazon basin hosts the most species-rich fish fauna in the world [1]. Historically, these aquatic environments have provided fish that help maintain ecological, cultural, and economic aspects of Amazonian livelihoods [2]. Additionally, some fish species, as the Piraíba (*Brachyplatystoma filamentosum*), the largest catfish of the Amazonian basin, contribute with protein food supply [2]. Worryingly, recent surveillance studies of antimicrobial resistance in aquatic environments of the Brazilian Amazon have revealed the presence of clinically relevant multidrug-resistant pathogens, including extended-spectrum β-lactamase (ESBL)-producing Enterobacterales [[3], [4], [5]], which have been recognized as critical-priority pathogens by the World Health Organization (WHO) [6]. In this study, we report for the first time, the identification and genomic features of a multidrug-resistant (MDR) ESBL-producing *Klebsiella pneumoniae* strain isolated from a native freshwater catfish commercialized for human consumption in a wet market of the Amazonian region of Brazil, denoting a hidden risk for the human health.

## 2 Materials and methods

In 2019, during a Brazilian surveillance study (OneBR project), conducted to characterize the burden of antimicrobial resistance associated with critical WHO priority pathogens [6], a broad-spectrum cephalosporin-resistant ESBL-producing *K. pneumoniae* strain (KpP1) was isolated from a Piraíba freshwater catfish sold in a local wet market in Belém city, northern Brazil. For microbiological analysis dissected specimens were aseptically collected from the anterior, middle, and hind region of the fish [7]. Samples were rinsed in 225 mL of sterile MacConkey broth and incubated at 37 °C for 24 h. After incubation, 1-mL aliquot of broth was serially diluted in buffered peptone water, inoculated onto MacConkey agar plates containing ceftriaxone (2 μg/mL) (Sigma-Aldrich, St. Louis, MO), and incubated at 37 °C for 24 h. Ceftriaxone-resistant colonies were identified by matrix-assisted laser desorption/ionization time-of-flight mass spectrometry (MALDI-TOF). Antimicrobial susceptibility was determined by Kirby-Bauer and *E*-test methods [8]. Genomic DNA was extracted using the PureLink™ Quick Gel Extraction kit (Life Technologies, Carlsbad, CA), according to the manufacturer's guidelines. Qubit 2.0 fluorometer (Life Technologies, Carlsbad, CA) was used to measure DNA concentration. Afterwards, the genomic library was prepared using the Nextera DNA Flex library preparation kit (Illumina, San Diego, CA) and, subsequently, sequenced using 2 × 75-bp paired-end library on a NextSeq550 platform (Illumina). Read with a PHRED quality score below 20 were discarded and adapters were trimmed using TrimGalore v0.6.5 (https://github.com/FelixKrueger/TrimGalore). *De novo* genome assembly was performed with Unicycler v0.4.8. [9]. Sequences were annotated using NCBI Prokaryotic Genome Annotation Pipeline (http://www.ncbi.nlm.nih.gov/genome/annotation_prok/) and Rapid Annotation System Technology (RAST) pipeline [10].

Plasmid replicon types were identified, *in silico*, using PlasmidFinder 2.1 (https://cge.cbs.dtu.dk/services/PlasmidFinder/). Disinfectant resistance genes and heavy metal resistance genes were detected using Pasteur database (http://bigsdb.pasteur.fr/klebsiella/klebsiella.html) and BacMet (http://bacmet.biomedicine.gu.se/), whereas Kleborate was used to screen assemblies to confirm the species designation, multilocus sequence type (ST), antibiotic resistance genes, ICE*Kp* associated virulence loci [yersiniabactin (*ybt*), colibactin (*clb*)], and K (capsule) and O antigen (LPS) serotypes [[11], [12], [13]]. For all predicted genes, a > 90% identity threshold was used as filter for identification. In addition, potential of antibiotic-resistance genes to be mobilized by the identified plasmids was analized by Machine Learning, which identified binary classification of contigs for plasmid- and chromosome-derived through mlplasmids tool [14].

## 3 Results and discussion

**Fig. 1:**
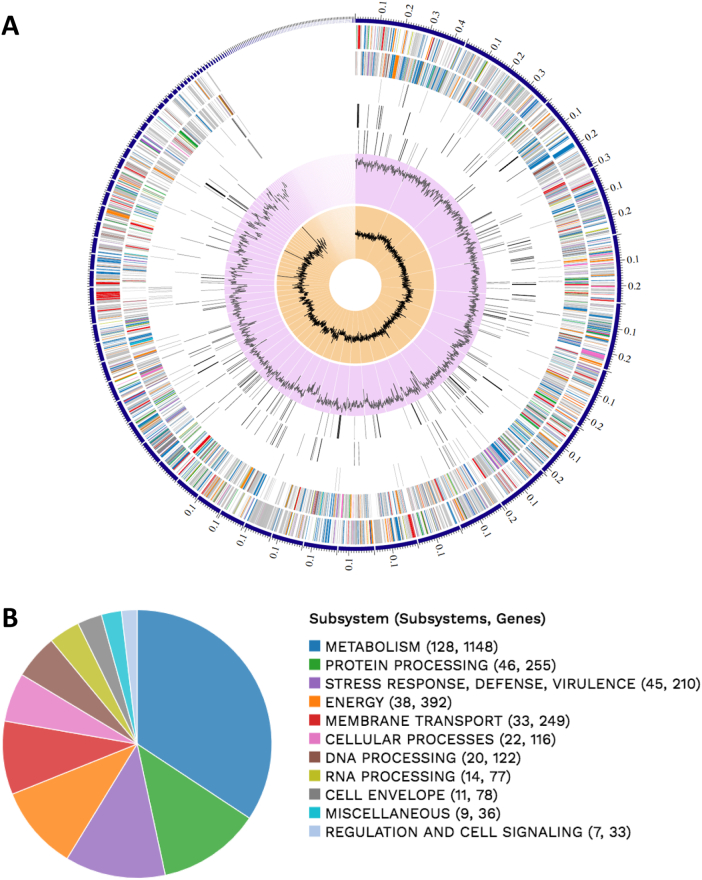
In A, circos plot, a circular graphical display of the distribution of the contigs of genome assembly. Genome annotations includes (from outer to inner rings): the contigs, CDS on the forward strand, CDS on the reverse strand, RNA genes, CDS with homology to known antimicrobial resistance genes, CDS with homology to know virulence factors, GC content and GC skew. The colors of the CDS on the forward and reverse strand indicate the subsystem that these genes belong to. In B, RAST functional annotation of *K. pneumoniae* KpP1 strain belonging to ST3827. Pie chart shows functional annotation of various subsystem genes.

Ceftriaxone-resistant lactose-positive colonies were recovered from meat samples of the anterior dissected section, being identified as *K. pneumoniae* (KpP1 strain). A multidrug-resistant profile to cephalothin, ceftriaxone (MIC>64 μg/mL), cefotaxime, aztreonam, tetracycline, sulfonamide, gentamicin, nalidixic acid (MIC>32 μg/mL) and ciprofloxacin (MIC>4 μg/mL); and susceptibility to carbapenems and colistin was confirmed. Assembly reports showed a total of 5715 genes with 5567 protein-coding sequences. A total of 111 contigs was obtained, with a N_50_ value of 172,388-bp, as well as G + C content of 56.7%. The genome of KpP1 was 5685.349-bp in size, containing 44 tRNAs, 3 rRNAs, 7 ncRNAs, 94 pseudogenes, and 1 CRISPR array. Circos plot and subsystem obtained from RAST are shown in Fig. 1A and B, respectively [15].

Whole-genome sequencing analysis revealed the presence of genes conferring resistance to β-lactams [*bla*_CTX-M-15_, *bla*_TEM-1B_, *bla*_SHV-27_], aminoglycosides [*aac(3)-IId*, *aadA1*], sulfonamides [*sul2*], tetracyclines [*tetB* and *tetD*], fosfomycin [*fosA*-like] and quinolones [*oqxA*, *oqxB, qnrE1*]. Interestingly, the identification of the plasmid-mediated quinolone resistance (PMQR) gene *qnrE1* has been restricted to South American countries within a One Health context [16]. While integrase and *qac* genes associated with class 1 integron were no identified, *oqxA/B* efflux pump genes associated with additional resistance to benzalkonium chloride and triclosan were predicted [17].

**Fig. 2:**
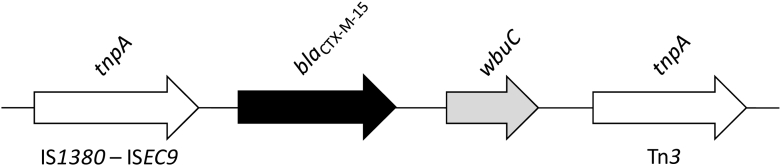
Schematic representation of the genetic context surrounding the *bla*_CTX-M-15_ gene in *K. pneumoniae* KpP1 strain isolated from a native Amazonian fish in Brazil. Arrows indicate the positions and directions of the genes.

The *bla*_CTX-M-15_ was located next to the *wbuC* gene (encoding a cupin fold-metalloprotein) and flanked upstream and downstream by an IS*Ec9* and a Tn*3* family transposase, respectively, commonly associated with CTX-M-15 genes (Fig. 2). Resistance genes for heavy metals, including silver (*silA-silS*), tellurite (*terA*, *terB*, *terC* and *terE*) and copper (*pcoA-pcoS*) were identified, as well as type 3 fimbriae (*mrkABCDFHIJ*) virulence genes, KL111 (*wzi*113) and O3b locus, and plasmid of incompatibility groups IncFII(K), IncFIB(mar) and IncHI1B. In this regard, mlplasmids showed that IncFII(K) carried *bla*_CTX-M-15_, whereas the hybrid-plasmid IncFIB(mar)/IncHI1B harbored the *qnrE1* gene, confirming that these plasmids play a role in spreading antimicrobial resistance, not only in clinical settings.

Finally, *K. pneumoniae* KpP1 strain was assigned to the sequence type (ST) 3827. Curiously, no epidemiological evidence for local or international spread of ST3827 was found, which could support the hypothesis of acquisition of clinically relevant resistance genes by autochthonous microbiota of local ecosystems in the Amazonian region, most likely associated with anthropogenic contamination of the Amazon basin hosting fish fauna [3,4]. In fact, KpP1 was genotyped by Kaptive as KL111, a K-locus different than virulence-associated KL1 and KL2 *K. pneumoniae* strains [13].

Pollution of aquatic environments, mainly by aquaculture, or from household, agricultural, industrial and hospital discharges have contributed for the spread of resistant bacteria and resistance genes worldwide [6,18]. On the other hand, the wet market itself could be a source of critical-priority WHO pathogens, since MDR bacteria could come from poor handling, storage and transportation practices that may predispose freshwater fish to contamination, which pose a food safety hazards. In this regard, CTX-M-producing *K. pneumoniae* strains have been increasingly reported in a variety of non-human sources, including food and food products, representing a serious threat to public health [19].

In summary, we present the first draft genome sequence of a MDR *K. pneumoniae* co-harboring *bla*_CTX-M-15_ and *qnrE1* genes, recovered from a freshwater fish. Our findings suggest that native freshwater fish sold in wet markets of the Amazonian region could be important vehicles for transmission of MDR bacteria to human. Therefore, this study may give a genomic insight on the spread of critical-priority WHO pathogens in a One Health context.

## Nucleotide sequence accession number

This Whole Genome Shotgun project has been deposited at DDBJ/ENA/GenBank under the accession JAAQON000000000. The version described in this paper is version JAAQON000000000.1. Additionally, genomic data of *K. pneumoniae* KpP1 strain is available on the OneBR platform under the number ID ONE302 (http://onehealthbr.com/).
